# Molecular and cellular origins of behavioral sex differences: a tiny little fly tells a lot

**DOI:** 10.3389/fnmol.2023.1284367

**Published:** 2023-10-16

**Authors:** Kosei Sato, Daisuke Yamamoto

**Affiliations:** Neuro-ICT Laboratory, Advanced ICT Research Institute, National Institute of Information and Communications Technology, Kobe, Japan

**Keywords:** Drosophila, fruitless, doublesex, terminal selectors, neural sexual dimorphism

## Abstract

Behavioral sex differences primarily derive from the sexually dimorphic organization of neural circuits that direct the behavior. In *Drosophila melanogaster*, the sex-determination genes *fruitless* (*fru*) and *doublesex* (*dsx*) play pivotal roles in producing the sexual dimorphism of neural circuits for behavior. Here we examine three neural groups expressing *fru* and/or *dsx*, i.e., the P1 cluster, aSP-f and aSP-g cluster pairs and aDN cluster, in which causal relationships between the dimorphic behavior and dimorphic neural characteristics are best illustrated. aSP-f, aSP-g and aDN clusters represent examples where *fru* or *dsx* switches cell-autonomously their neurite structures between the female-type and male-type. Processed sensory inputs impinging on these neurons may result in outputs that encode different valences, which culminate in the execution of distinct behavior according to the sex. In contrast, the P1 cluster is male-specific as its female counterpart undergoes *dsx*-driven cell death, which lowers the threshold for the induction of male-specific behaviors. We propose that the products of *fru* and *dsx* genes, as terminal selectors in sexually dimorphic neuronal wiring, induce and maintain the sex-typical chromatin state at postembryonic stages, orchestrating the transcription of effector genes that shape single neuron structures and govern cell survival and death.

## Introduction: studying fly mating unveils the logic of brain circuit rearrangements

Behavioral sex difference is widespread in the animal kingdom. The most conspicuous cases are often found in mating behavior, where a female and a male are driven by conflicting interests for their own reproductive success, yet cooperation between the two individuals is indispensable for successful reproduction. Faced with such conflicting demands, selective pressure will act to exaggerate the dimorphic traits in both morphology and behavior (Price et al., 2023; Tosto et al., 2023). Our understanding of the genetic and cellular mechanisms for morphological sexual differences has been fueled by the rapid grow of the evo-devo field, but the mechanistic basis of behavioral sexual dimorphisms remains enigmatic, primarily because the complex architecture of neural circuits underlying behaviors has hampered the cell-by-cell analysis of dimorphic connectivity.

*Drosophila melanogaster* provides a suitable experimental system for unraveling the neural substrates of dimorphic behaviors, because sophisticated genetic techniques for labeling and manipulation of neurons are available, in conjunction with applicability of electrophysiology and Ca^2+^-imaging for neural activity recordings in live, behaving animals, allowing one to identify the neuron that plays a key role in the expression of dimorphic behaviors (Yamamoto, 2022). Since *D. melanogaster* is one of only a few species in which the whole brain connectome has been completed (Scheffer et al., 2020; Dorkenwald et al., 2021), the neurons found to be important for a particular behavior can immediately be mapped in the deduced connectomic circuits.

Indeed, the circuits for sociosexual behaviors such as courtship and aggression have been particularly well characterized in *D. melanogaster* (Koganezawa et al., 2016; Asahina, 2017; Chiu et al., 2021; Jiang and Pan, 2022), since two sex determination genes *fruitless* (*fru*) and *doublesex* (*dsx*) play instructing roles for the configuration of the sex-specific circuit, often referred to as the *fru/dsx*-circuit (Cachero et al., 2010; Yu et al., 2010), and consequently, the molecular and genetic resources derived from these two genes (e.g., a large array of transgenic and mutant flies and specific antibodies) offer an unparalleled opportunity to selectively manipulate the sex-specific circuit with a minimal disturbance of other neural networks in the brain (Demir and Dickson, 2005; Rideout et al., 2010; Neville et al., 2021; Sato and Yamamoto, 2022).

The organization and function of the *fru/dsx*-circuit has been reviewed several times (Yamamoto and Koganezawa, 2013; Coen and Murthy, 2016; Ellendersen and von Philipsborn, 2017) and we do not intend to repeat a general overview of this circuit. Instead, this article focuses on a few select studies that successfully unveiled how a specific cellular sex difference in an identified neuron could lead to a clear sex difference in certain behavior. We also attempt to construct a coherent framework in which *fru/dsx* gene products reconfigure subcellular structures of a neuron so that the neuron is incorporated into a female-typical or male-typical circuit that produces a sex-specific behavior.

## Male-specific P1 neurons: are they essential for executing male courtship?

The P1 neuron cluster is composed of (per hemibrain) 20 male-specific neurons that express both the male-specific *fru* product FruM and the male-specific *dsx* product DsxM (Kimura et al., 2008). It is a prevalent view that P1 neurons are essential for males in initiating courtship behavior toward a female. In fact, P1 neurons were discovered by an unbiased screen of mosaic females that carry tens of masculinized *fru* expressing neurons for the ability to court another female with a male-typical posture, i.e., unilateral wing vibration: the P1 cluster was the sole masculinized neuron group that was found significantly more often in courtship-positive females than courtship-negative females (*p* < 0.0001 by Fisher’s exact probability test; Kimura et al., 2008). In other words, a few mosaic females did not have a masculinized P1 cluster, but they nonetheless displayed male-typical courtship behavior. This initial observation already indicated that the presence of P1 neurons is not essential for the initiation of male courtship (even in mosaic females). This important point was thoroughly discussed in a review article by Siwicki and Kravitz (2009) that appeared soon after the publication of the original article. As a matter of fact, 5 months prior to the report of Kimura et al. (2008) and Clyne and Miesenböck (2008) reported that decapitated females were able to generate a quasi-normal courtship (pulse) song when the neurons in the ventral nerve cord were artificially activated via P2X_2_, although the temporal structure of the song was distorted. An even more striking observation was made by Demir and Dickson (2005), who demonstrated that females that transgenically express the male-specific FruM display male-typical courtship toward other female flies. Notably, these FruM-overexpressing females do not have P1 neurons, because they are females and FruM has nothing to do with the presence or absence of P1 neurons (see below; Kimura et al., 2008). However, we found that the courting activity of these females that lack P1 neurons was very low. In male flies, thermogenetic or optogenetic activation of P1 neurons readily induced courtship pursuit particularly when such males were, at the same time, pheromonally stimulated, or visually stimulated by not only a moving female but also a moving dummy or even moving stripes displayed on a computer screen (Kohatsu et al., 2011; Pan et al., 2012; Kohatsu and Yamamoto, 2015; see also Agrawal et al., 2014 for the role of vision). P1 neurons were shown to respond to these chemical and visual stimuli with Ca^2+^ rises, which were sustained beyond the stimulation period under constrained conditions (Inagaki et al., 2014) or recurrently occurred while the male pursued a courtship target (Kohatsu and Yamamoto, 2015). These observations collectively indicate that P1 neurons are dispensable for generating a series of courtship actions. Rather, P1 neurons seem to boost up male courtship activity and increase the coherence among distinct courtship actions and time organizations. Therefore, the presence or absence of P1 neurons does not produce a qualitative difference between the female and male courtship behavior, but P1 neuron activities increase the level and fidelity of male courtship behavior.

## aSP-f and aSP-g: changeover switch of behavioral sex types in a pheromone pathway

**Figure 1:**
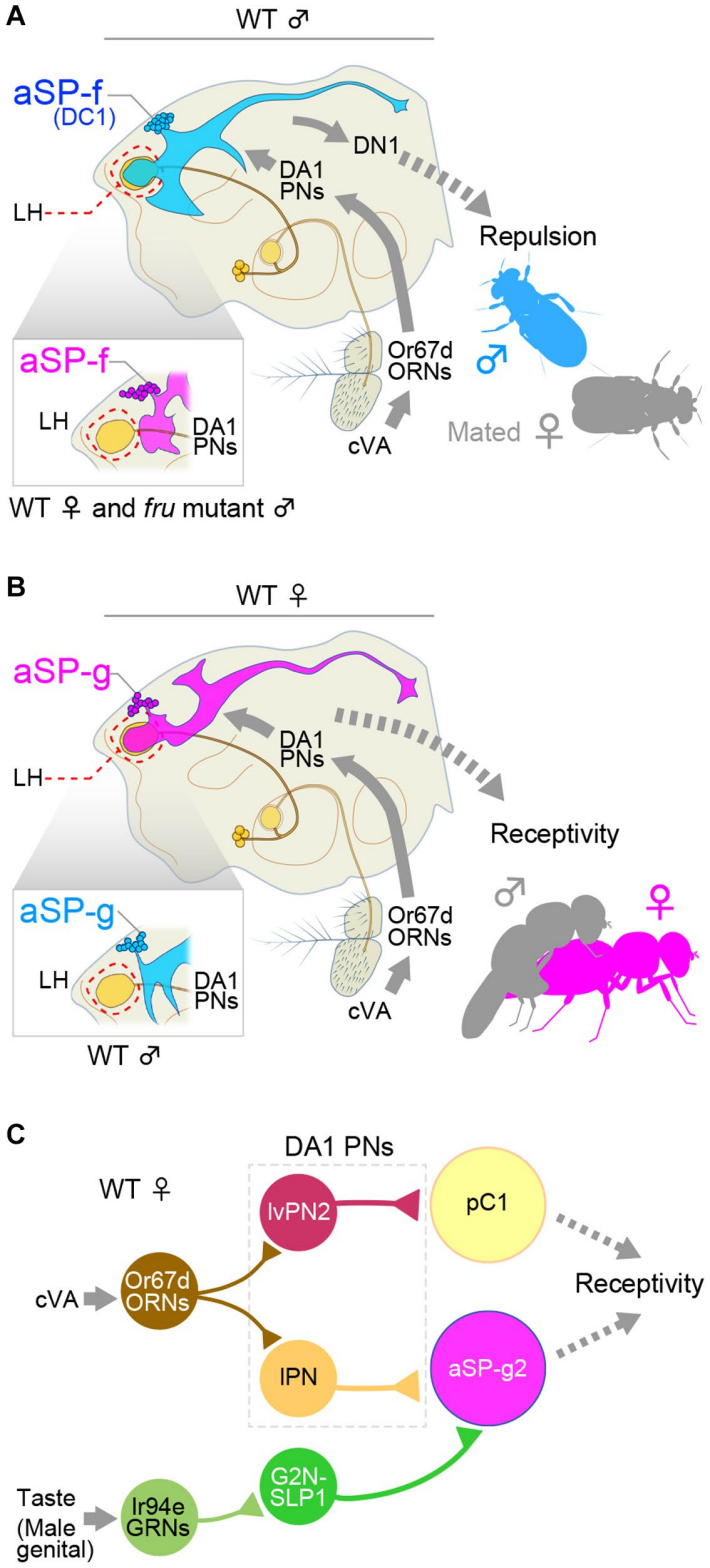
*fru*-expressing aSP-f and aSP-g interneurons form a changeover switch in the pheromone *cis*-vaccenyl acetate (cVA)-processing pathways. **(A)** cVA activates the Odorant receptor 67d (Or67d)-expressing sensory neurons (ORNs) in the antenna, which project to the DA1 glomerulus. DA1 projection neurons (PNs) send their axon to the lateral horn (LH), where they synapse onto aSP-f (DC1) interneurons, which, in turn, connect with a descending interneuron, DN1, in males. **(B)** In females, DA1 projection neurons synapse onto aSP-g interneurons without forming synapses onto aSP-f interneurons. This bidirectional circuit switch reroutes the cVA signals to different descending neurons, resulting in the distinct behavioral outputs in males and females. **(C)** A diagram of the circuit integrating olfactory and gustatory information, which controls female receptivity. lPN, lateral projection neuron; lvPN, lateroventral projection neuron; GRNs, gustatory receptor neurons; G2N-SLP1, gustatory second order neuron-superior lateral protocerebrum. Panel **(C)** was drawn based primarily on the results reported by Kohl et al. (2013) and Taisz et al. (2023).

*cis*-vaccenyl acetate (cVA) is synthesized in the male ejaculatory bulb and transferred to the female uterus with seminal fluid (Brieger and Butterworth, 1970; Guiraudie-Capraz et al., 2007), attenuating attractiveness of the female as a mating partner for males; it thus acts as a mating inhibitory pheromone for males (Siegel and Hall, 1979). However, for females, and even for males under certain conditions, cVA is attractive and induces aggregation (Bartelt et al., 1985; Cortot et al., 2022). The neural basis for the sexually dimorphic actions of cVA has been successfully tracked back to two identified clusters of neurons, aSP-f (also known as DC1; Figure 1A) and aSP-g (Figure 1B), which are third-order interneurons in the olfactory center lateral horn (Kohl et al., 2013). Patch clamp recordings from MARCM clone neurons revealed that male aSP-f and female aSP-g receive monosynaptic inputs from DA1 projection neurons, which relay cVA signals originating from antennal sensory neurons that express Odorant receptor 67d (Or67d) (Datta et al., 2008) to the lateral horn via the sexually dimorphic DA1 glomerulus of the antennal lobe (Kondoh et al., 2003; Stockinger et al., 2005). aSP-f/DC1 and aSP-g neurons each send output to different postsynaptic neurons, so that the behavioral outcome of sensing cVA becomes distinctly different between the sexes—i.e., attraction in females and repulsion in males (Kohl et al., 2013). Loss of FruM in mutant MARCM clones in male flies transforms the dendritic field of these two neural types: aSP-f/DC1 loses, while aSP-g acquires, morphological and electrophysiological connections with DA1 projection neurons. Thus, FruM masculinizes the dendritic patterns of aSP-g and aSP-f/DC1, allowing otherwise non-sex-specific cVA sensory input drives either female-specific or male-specific behavior depending on the fly’s sex (Kohl et al., 2013). In this case, a single transcription factor (FruM) switches the information flow in the brain through its action on neurite patterning according to the sex.

aSP-f/DC1 in males, then, activates the male-specific descending neuron DN1, although the behavioral outcome of this activation has not been elucidated (Ruta et al., 2010). Additionally, a tachykinin-expressing subpopulation of aSP-f in males was reported to promote same-sex aggression (Asahina et al., 2014). On the other hand, a recent work gave a new twist on the behavioral roles of aSP-g in females (Figure 1C). Taisz et al. (2023) distinguished three morphological subtypes of aSP-g, and showed that only the aSP-g2 population possesses a dendritic field that overlaps with the DA1 projection neuron terminals. Connectome data suggested that aSP-g2 is likely to be postsynaptic to a gustatory interneuron named G2N-SLP1 (an abbreviation of gustatory second order neuron-superior lateral protocerebrum). Taisz et al. (2023) reported that G2N-SLP1 conveys, to aSP-g2, input from Ir94e-expressing labellar gustatory receptor neurons (Ir94e-GRNs), which they found to respond strongly to male genitals, in addition to their known ligands, water and NaCl (Jaeger et al., 2018). Intriguingly, optogenetic activation of both Ir94e-GRNs and c-VA-sensitive olfactory projection neurons, but not either one of the two, promoted female acceptance, and optogenetic activation of aSP-g2 alone recapitulated the effect of gustatory and olfactory dual stimulations (Taisz et al., 2023). These observations elegantly documented that a dendritic sexual dimorphism of a specific neural node offers a decisive mechanism whereby the same sensory input triggers radically different behavioral outputs between the two sexes.

## The aDN cluster: crossing the border between visual and olfactory perception

**Figure 2:**
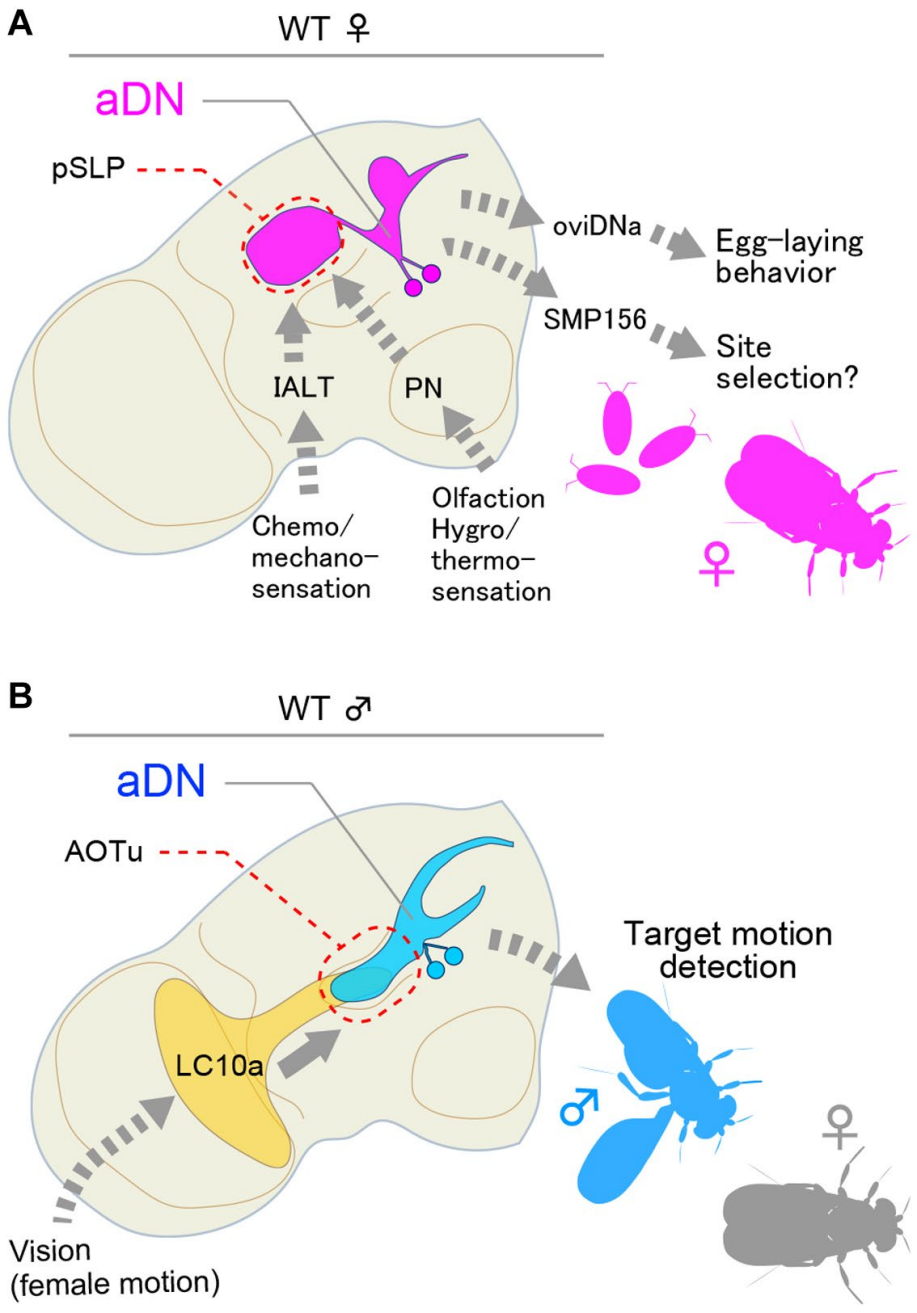
*dsx*-expressing aDN neurons receive sexually dimorphic input and generate distinct behavior in females and males. **(A)** Female aDN neurons receive multimodal sensory input in the posterior part of the superior lateral protocerebrum (pSLP), superior clamp (SCP), and ventrolateral protocerebrum (VLP) with predominating olfactory input from the lateral horn for the control of communal egg-laying behavior. **(B)** Male aDN neurons extend their neurite in the anterior optic tubercle (AOTu), where they receive visual input from LC10a visual projection neurons. These neurons regulate visual courtship pursuit toward a female. IALT, lateral antennal lobe tract.

The aDN cluster composed of two pairs of neurons that express *dsx* but not *fru* exhibits a sexual dimorphism in neurite distributions and plays important roles in both sexes but in different behavioral contexts: in females, aDN contributes to oviposition site selection (Figure 2A), whereas, in males, it mediates visually guided courtship orientation (Figure 2B; Nojima et al., 2021). This functional diversification of aDN between the sexes was found to stem primarily from a difference in its presynaptic partners (Nojima et al., 2021): male aDN receives input from LC10a visual interneurons (Ribeiro et al., 2018) in the anterior optic tubercle (AOTu), and female aDN receives multimodal sensory input in the posterior part of the superior lateral protocerebrum (pSLP), superior clamp (SCL) and ventrolateral protocerebrum (VLP) with predominating olfactory input from the lateral horn (LH). Indeed, inactivation of aDN induced misdirected orientation in courting males and loss of preference for the medium deposited with male pheromone as an oviposition site in mated females (Nojima et al., 2021). In contrast to the diverged dendritic fields, the output site of aDN remained nearly the same in the two sexes, i.e., the superior medial protocerebrum (SMP). Connectome data indicated that the predominating output neuron for female aDN is the uncharacterized SMP156 neuron, which forms an “efferent” path from the SMP to the lobula in the contralateral optic lobe (Nojima et al., 2021). It remains to be determined whether male aDN similarly connect with a SMP homolog, forming a recurrent path to the lobula. If this is the case, the feedback loop will presumably be used by the male to correct errors in orienting a visual target (Shadmehr et al., 2010). By analogy, olfaction-based navigation of a female fly to a possible oviposition site might be fine-tuned by the SMP efferent path. Thus, this study appears to unveil a deep homology in the logic underlying circuit architecture for two types of navigation, one relying on visual input operating for male-specific behavior and the other relying on olfactory input operating for female-specific behavior. This also represents an economical solution for switching behavioral machineries between the sexes, which can be attained by a simple change in dendritic positioning within just two pairs of neurons as instructed by one sex-determination gene, *dsx*.

## Closing remarks

In the above sections, we examined three remarkable findings that illuminate specific cellular changes in the neural circuit that are causally related to sex differences in behavior. All cellular changes observed in these studies occurred cell autonomously within single neurons that express *fru* and/or *dsx*. Then, how do *fru* and *dsx* orchestrate such a major set of changes? Because the products of *fru* and *dsx* genes are transcription factors, the diversity in their functions likely derives from the diversity of their transcriptional targets (Luo et al., 2011; Ito et al., 2012; Clough et al., 2014; Neville et al., 2014; Ghosh et al., 2019; Li et al., 2022; Palmateer et al., 2023).

P1 neurons express both *fru* and *dsx* and are male-specific because female P1 homologs are fated to die as directed by the female-type *dsx* product DsxF (Kimura et al., 2008). However, females have a large set of P1-like neurons, PC1, which play central roles in aggression and receptivity control (Zhou et al., 2014; Deutsch et al., 2020; Chiu et al., 2021; Wang et al., 2021).

Some *fru*-single positive neuron clusters contain different numbers of neurons depending on the sex. For example, the mAL cluster is composed of 5 and 30 cells in the female and male brain, respectively (Kimura et al., 2005). This difference in cell number between the sexes arises from both sexually dimorphic proliferation and cell death, suggesting that *dsx* and possibly *fru* gene products regulate these fundamental cellular processes (Kimura et al., 2005; Ren et al., 2016; Ghosh et al., 2019).

We saw above that third-order processing of cVA information involves aSP-f in males and aSP-g in females, and the switching of synaptic partners is accomplished by the extension and retraction of dendritic branches by these neurons, which in turn depend on whether the neurons contain FruM (Kohl et al., 2013). In a similar manner, aDN switches its synaptic partners from/to olfactory vs. visual interneurons dependent on the presence or absence of DsxM (Nojima et al., 2021). In addition, P1 neurons extend a neurite in unusual directions in the male brain when FruM is lost from the P1 neurons themselves in the otherwise *fru*^+^ wild-type mosaic brain (Kimura et al., 2008). These observations collectively suggest that transforming dendritic branches at a hub in information flow is one of the major mechanisms for generating dimorphic circuits for sex-specific behavior by FruM and Dsx proteins. In fact, two firmly established transcriptional targets for FruM are two guidance molecules, i.e., *robo1* and *tei* (Ito et al., 2016; Sato et al., 2020). Õzel et al. (2022) proposed that the identity of individual neurons determined early in life is implemented in mature neurons by a neuron-specific code (the terminal selector code; Hobert and Kratsios, 2019) made up of ~10 transcriptional factors, whose sustained expression maintains the identity throughout the postembryonic stage. They showed in the *Drosophila* optic lobe that gain or loss of just one transcription factor alters the code so that the manipulated neuron transforms into another type of neuron by changing, for example, its dendritic pattern. The terminal selector transcription factors directly regulate the transcription of genes for effector proteins, such as *Dscam4* and the Netrin receptor *frazzled* (Õzel et al., 2022). Expression of FruM and Dsx is sustained throughout the adult stage (Lee et al., 2000; Usui-Aoki et al., 2000) and directly regulates the transcription of effector proteins including *robo1* and *tei* in the case of FruM (Ito et al., 2016; Sato et al., 2020). It is thus tempting to hypothesize that FruM and Dsx contribute to terminal selector codes for sex-specific neuronal characteristics required for generating sex-typical behaviors. FruM and probably also Dsx form a large complex with several other transcription factors to remodel chromatin structures for orchestrating transcription of a large number of target genes to shape sex-specific neuronal structure and function (Ito et al., 2012, 2016; Neville et al., 2014; Vernes, 2014; Chowdhury et al., 2017; Zhang et al., 2018; Sato et al., 2019, 2020). A recent snRNA-seq analysis in the sexually dimorphic bed nucleus of stria terminalis of the mouse brain unraveled a similar genome-wide chromatin rearrangement triggered by estrogen receptor activation for sex-specific network patterning in the perinatal stage, which represents the organizational effect of sex steroids (Gegenhuber et al., 2022). Mammalian sex steroids also mediate the manifestation of secondary sex characteristics and sexual drive after adolescence, demonstrating the activational effect of sex steroids. A deep homology between mammalian and insect neural sex differentiation processes implies the existence of adult-specific effects of FruM and Dsx, in addition to their roles in development. Indeed, Chen et al. (2021) proposed that FruM expressed in adult neurons inhibits male-to-male courtship, while pupal FruM instructs the circuit formation for male-to-female courtship behavior, based on the distinctly different effects of *fru* knockdown at the adult vs. pupal stages. Notably, pheromone-sensitive Or47b-expressing olfactory neurons exhibit experience-dependent FruM expression and functional modulation at the adult stage (Hueston et al., 2016; Sethi et al., 2019). There exists evidence that the P1a subset of P1 neurons, which plays a critical role in male-to-female courtship, may be dispensable for inducing male–male courtship (Bonheur et al., 2023).

This review article primarily focused on sexually dimorphic shaping of synaptic connectivity and survival of specific neuron types via cell autonomous actions of putative terminal selector transcription factors. Attention must be paid, however, to the fact that non-cell autonomous actions of a terminal selector transcription factor also play pivotal roles in organizing sexually dimorphic circuits. Indeed, an array of neuropeptides have been shown to modulate outputs of the *fru* circuit depending on the behavioral context as paracrine or endocrine signals (Lee et al., 2006; Kim et al., 2013; Asahina et al., 2014; Chen et al., 2017). Of note, some of *fru* developmental roles may be mediated by cell non-autonomous actions via cell-to-cell interactions, which affect survival and morphogenesis of synaptic partners (Brovero et al., 2021). Although the *fru*- and/or *dsx*-expressing neurons that process the pheromone inputs were the major subject of this article, these transcription factors are vital for sex-specification of neurons in the motor pathways (e.g., premotor neurons involved in copulation control; Crickmore and Vosshall, 2013; Kim et al., 2013).

The principle of circuit rearrangements by a sex-specific terminal selector in development may apply to sex-type specification in the mammalian brain, in which sex steroids initiate the entire process.

Further studies are needed to validate the hypothesis that the sex-specific terminal selector code acts throughout life to accomplish innate and experience-dependent wiring for gendered behavior across the animal kingdom.

## Author contributions

KS: Funding acquisition, Writing – original draft. DY: Conceptualization, Funding acquisition, Writing – original draft, Writing – review & editing.
